# Cerebrovascular pressure reactivity monitoring using wavelet analysis in traumatic brain injury patients: A retrospective study

**DOI:** 10.1371/journal.pmed.1002348

**Published:** 2017-07-25

**Authors:** Xiuyun Liu, Joseph Donnelly, Marek Czosnyka, Marcel J. H. Aries, Ken Brady, Danilo Cardim, Chiara Robba, Manuel Cabeleira, Dong-Joo Kim, Christina Haubrich, Peter J. Hutchinson, Peter Smielewski

**Affiliations:** 1 Division of Neurosurgery, Department of Clinical Neurosciences, Addenbrooke’s Hospital, University of Cambridge, Cambridge, United Kingdom; 2 Institute of Electronic Systems, Warsaw University of Technology, Poland; 3 Department of Intensive Care, University of Maastricht, Maastricht University Medical Center, Maastricht, The Netherlands; 4 Baylor College of Medicine, Houston, Texas, United States of America; 5 Department of Neuroscience, University of Genova, Genova, Italy; 6 Department of Brain & Cognitive Engineering, Korea University, Seoul, South Korea; 7 Faculty of Medicine, University of Aachen, Germany; Oregon Health and Science University, UNITED STATES

## Abstract

**Background:**

After traumatic brain injury (TBI), the ability of cerebral vessels to appropriately react to changes in arterial blood pressure (pressure reactivity) is impaired, leaving patients vulnerable to cerebral hypo- or hyperperfusion. Although, the traditional pressure reactivity index (PRx) has demonstrated that impaired pressure reactivity is associated with poor patient outcome, PRx is sometimes erratic and may not be reliable in various clinical circumstances. Here, we introduce a more robust transform-based wavelet pressure reactivity index (wPRx) and compare its performance with the widely used traditional PRx across 3 areas: its stability and reliability in time, its ability to give an optimal cerebral perfusion pressure (CPPopt) recommendation, and its relationship with patient outcome.

**Methods and findings:**

Five hundred and fifteen patients with TBI admitted in Addenbrooke’s Hospital, United Kingdom (March 23rd, 2003 through December 9th, 2014), with continuous monitoring of arterial blood pressure (ABP) and intracranial pressure (ICP), were retrospectively analyzed to calculate the traditional PRx and a novel wavelet transform-based wPRx. wPRx was calculated by taking the cosine of the wavelet transform phase-shift between ABP and ICP. A time trend of CPPopt was calculated using an automated curve-fitting method that determined the cerebral perfusion pressure (CPP) at which the pressure reactivity (PRx or wPRx) was most efficient (CPPopt_PRx and CPPopt_wPRx, respectively).

There was a significantly positive relationship between PRx and wPRx (r = 0.73), and wavelet wPRx was more reliable in time (ratio of between-hour variance to total variance, wPRx 0.957 ± 0.0032 versus PRx and 0.949 ± 0.047 for PRx, *p* = 0.002). The 2-hour interval standard deviation of wPRx (0.19 ± 0.07) was smaller than that of PRx (0.30 ± 0.13, *p* < 0.001). wPRx performed better in distinguishing between mortality and survival (the area under the receiver operating characteristic [ROC] curve [AUROC] for wPRx was 0.73 versus 0.66 for PRx, *p* = 0.003). The mean difference between the patients’ CPP and their CPPopt was related to outcome for both calculation methods. There was a good relationship between the 2 CPPopts (r = 0.814, *p* < 0.001). CPPopt_wPRx was more stable than CPPopt_PRx (within patient standard deviation 7.05 ± 3.78 versus 8.45 ± 2.90; *p* < 0.001).

Key limitations include that this study is a retrospective analysis and only compared wPRx with PRx in the cohort of patients with TBI. Prior prospective validation is required to better assess clinical utility of this approach.

**Conclusions:**

wPRx offers several advantages to the traditional PRx: it is more stable in time, it yields a more consistent CPPopt recommendation, and, importantly, it has a stronger relationship with patient outcome. The clinical utility of wPRx should be explored in prospective studies of critically injured neurological patients.

## Abstract 2

Using continuous monitoring data in traumatic brain inury patients, Xiuyun Liu and colleagues compare the performance of cerebrovascular pressure reactivity monitoring using wavelet analysis to the pressure reactivity index.

## Author summary

**Why was this study done?:**

- The brain is vulnerable to damage from too little (ischemia) or too much (hyperemia) blood flow following traumatic brain injury (TBI).
- A physiological mechanism called cerebral autoregulation (CA) exists to maintain stable blood flow even if cerebral perfusion pressure (CPP) is changing, and an assessment of CA as part of bedside neuro-monitoring of patients with TBI could facilitate individualized treatment.
- A robust method for assessing CA in TBI is not yet available. The traditional measure used, the pressure reactivity index (PRx), provides inherently noisy estimates and may not be reliable in various clinical circumstances.

**What did the researchers do and find?:**

- We studied a new method to assess cerebral CA using continuous, real-time assessment of CA from available neuro-monitoring data called transform-based wavelet pressure reactivity index (wPRx).
- Using data collected from the continuous monitoring of arterial blood pressure (ABP) and intracranial pressure (ICP) from 515 patients with TBI, we compared the performance of wPRx with PRx across 3 areas: its stability/reliability in time, its ability to give an optimal cerebral perfusion pressure (CPPopt) recommendation, and its relationship with patient outcome.
- We found a significant positive relationship between PRx and wPRx, with wPRx being more stable in time.
- wPRx performed better in distinguishing different patient outcome groups, while CPPopt calculated using wPRx had lower variance and higher overall yield (percent of time that the value could be calculated).

**What do these findings mean?:**

- In this study, we found wPRx to be more stable in time and yield a more consistent CPPopt recommendation compared to PRx. Importantly, wPRx had a stronger relationship with patient outcome compared to PRx in our study.
- The clinical utility of wPRx should be explored further in prospective studies of critically injured neurological patients, particularly in circumstances in which reliability of PRx has been questioned, e.g., inpatient after decompressive craniectomy.

## Introduction

Traumatic brain injury (TBI) is an important global public health problem as a major cause of traumatic death and disability, especially in young people [1,2]. Management of these critically ill patients hinges on the early identification of secondary injuries to the brain and typically involves monitoring of intracranial pressure (ICP) and cerebral perfusion pressure (CPP). With increased sophistication of neuro-monitoring techniques, there is now the opportunity to interrogate cerebral homeostatic mechanisms that may represent hitherto underrecognized pathophysiological pathways [3]. One such cerebral homeostatic mechanism is the ability of the brain to maintain constant cerebral blood flow despite variations in CPP—a mechanism known as cerebral autoregulation (CA) [4]. Failure of this defensive mechanism, even for a short period, may lead to serious consequences, particularly in vulnerable patients following TBI. Therefore, monitoring of CA has been proffered as beneficial for the detection of secondary brain injuries and potentially has a therapeutic role in management of severe TBI [5].

Over the past 2 decades, the clinical assessment of CA has transitioned from intermittent testing to continuous monitoring [4,6–9]. Czosnyka et al. proposed that the relationship between the spontaneous, slow changes in mean arterial blood pressure (ABP) and ICP may shed light on the relationship between CPP and cerebral blood flow (CA), giving rise to the development of the pressure reactivity index (PRx) [10]. PRx is calculated at the bedside simply as the Pearson correlation coefficient between the slow changes in ABP and ICP over a short window of time (e.g., 5 minutes), updated every minute to yield continuous monitoring. For the case of impaired cerebral pressure reactivity, we would expect that an increase in intraluminal ABP will cause passive cerebrovascular dilation, resulting in increases in cerebral blood volume and thus ICP. In this situation, the correlation coefficient between ABP and ICP will be positive; that is, the PRx will be close to +1. Conversely, if cerebral pressure reactivity is intact, slow increases in ABP will cause decreases in ICP—PRx will be negative.

The clinical utility of PRx in patients with severe TBI has been demonstrated since its development. It can help predict patient outcome; alert the clinician to subtle cerebrovascular impairment or the response to various common clinical interventions [11,12]; and finally, with the help of curve fitting, it can be used to continuously estimate the optimal CPP (CPPopt) [13–15] by plotting PRx against CPP, which generally produces a U-shape curve—with CPP value at the minimum PRx associated with the strongest autoregulatory ability [14]—and thus can help direct patient management. However, the traditional PRx has several disadvantages: it is an inherently noisy parameter, it cannot precisely target specific frequencies at which pressure reactivity might be operating, and it assumes that the relationship between ABP and ICP does not change within the calculation period, i.e., it assumes a stationary process [16].

By contrast, the wavelet transform method, which has been widely applied in the field of geophysics and economics, has been found to be particularly useful for analyzing intermittent, noisy, and nonstationary signals [17], making it an ideal candidate for assessing cerebral pressure reactivity. The 2 parameters that are typically used to characterize wavelet analysis are the wavelet phase shift, which measures the delay between the 2 signals over a range of frequencies and time points [18], and the wavelet coherence, which characterizes cross-correlations between 2 signals [19].

In this study, we introduce wavelet transform analysis to assess cerebral pressure reactivity in 515 patients with TBI. The wavelet method was compared with the traditional parameter, PRx, in terms of pragmatic benchmarks: its stability and reliability, its inherent prognostic information, and its ability to estimate CPPopt.

## Methods

### Ethics

The data analyzed in this study consist of 2 groups. One was reviewed and approved by the local ethics committee of Addenbrooke’s Hospital, Cambridge University, United Kingdom (30 REC 97/291), and the other includes anonymized routine clinical data not collected under patient/next of kin consent/assent, in accordance with UK legislation. The Research Governance at Cambridge University Hospitals NHS Trust approved the use of the data for this and other methodological studies since the data have been anonymized, and, in such cases, there was no requirement from NHS Digital for consent to be obtained. This study is reported as per the Strengthening the Reporting of Observational Studies in Epidemiology (STROBE) Statement (S1 Checklist). We followed the prospectively written research plan document for the cohort studies (S1 Research Plan).

### Data sample

This study included a total of 515 patients with TBI who had continuous monitoring of ABP and ICP and were admitted to Addenbrookes’ Hospital, University of Cambridge, UK, between March 23rd, 2003 to December 9th, 2014. ABP was monitored invasively through the radial or femoral artery using a standard pressure monitoring kit (Baxter Healthcare, CardioVascular Group, Irvine, CA). ICP was monitored using an intraparenchymal probe (Codman ICP MicroSensor, Codman & Shurtleff, Raynham, MA) inserted into the frontal cortex [14]. All signals were sampled at 30–240 Hz (the sampling rates were increased over the years) and recorded using ICM+ software (University of Cambridge, Cambridge Enterprise, Cambridge, UK, http://www.neurosurg.cam.ac.uk/icmplus) through an A/D converter (DT9801, Data Translation, Marlboro, MA) or digitally directly from GE Solar monitors. The preanalysis, including artifacts removal, data trend calculation, and all the CA parameters (PRx, wavelet pressure reactivity index [wPRx]) were calculated through ICM+ software. Artifacts introduced by tracheal suctioning, arterial line flushing, or transducer malfunction were removed manually. Inclusion criteria were all patients with TBI, with availability of ABP and ICP monitoring,. There were no explicit exclusion criteria; however, some of the patients from that time period may have been omitted due to technical/staffing reasons of high-resolution data collection process. Patients were managed according to current institutional TBI guidelines (adapted from Menon et al., 1999) [20]. In brief, patients were sedated, intubated, ventilated, and in some cases, paralyzed. CPP was managed according to ICP/CPP management protocol. Interventions were aimed at keeping ICP <20 mmHg using positioning, sedation, muscle paralysis, moderate hyperventilation, ventriculostomy, osmotic agents, and induced hypothermia. CPP was maintained above 60 to 70 mmHg using vasopressors, inotropes, and intravenous fluids. CA assessment parameters analyzed in this study were not included in patients’ management; therefore, the clinical outcome was independent, and the analysis of patient outcome with these CA parameters was valid.

### Data analysis

#### CA parameters

Time-averaged values of ICP, ABP, and CPP (CPP = ABP − ICP) were calculated using waveform time integration over 10-second intervals. PRx was calculated as a moving Pearson correlation coefficient between 10-second averages of ABP and mean ICP, using a 300-second data window [21].

#### Wavelet analysis

Wavelet transform phase shift (WTP), the phase difference between ABP and ICP, in the frequency of 0.0067 Hz to 0.05 Hz was calculated through complex wavelet transform, described in S1 Appendix [19,22–24]. Morlet mother wave with the central frequency at 1 Hz was applied either stretched or compressed to match various components of ABP/ICP signals (See S1 Appendix). A 500-second window was used to calculate WTP, in order to allow enough data points to be calculated after removing the edge effect (see Appendix) [19,25], and updated every 10 seconds. Wavelet transform coherence (WTC) was applied as an indicator of a reliable phase relationship between input and output, decided through Monte Carlo simulations approach. The simulations were conducted 10,000 times to generate the distribution of estimated WTC values of 2 uncorrelated signals [19,26]. If the actual value of WTC was higher than 95% of the WTC values obtained in this artificial, unrelated surrogate distribution, it was assumed to be high enough to indicate a significant relationship between input and output. Individual WTP values with WTC higher than the threshold were included, while the points with WTC lower than the threshold were ignored. The cosine of the WTP angle, termed wPRx, was calculated afterwards for 2 reasons: First, the phase angle is converted to a number between −1 (180 degrees phase shift) and +1 (0 degrees phase shift) that is directly comparable to the PRx. Second, the cosine operation offers a practical solution to the problem of phase wrapping, as it is effectively equivalent to the normalized real part of the complex wavelet cross-spectrum value.

#### Reliability and stability of PRx and wPRx

The stability of PRx and wPRx was tested by taking distinct time points. We selected 1 PRx/wPRx point every 10 minutes, then the standard deviation (SD) of these PRx/wPRx points over a period of 1 hour was calculated, i.e., SD of six numbers. The calculation was repeated every 2 hours, thus leaving a 1-hour gap between data segments. Subsequently, a paired comparison of the pulled data of 2-hour interval SD of PRx and wPRx was performed (*n* = 33,000).

The reliability of the PRx and wPRx was also tested by taking a distinct group of time points. Each patient’s data were split into hours, and in order to avoid the overlapping windows, we extracted wPRx (PRx) of a 1-hour period every 3 hours, with a 2-hour gap between data segments. The variance within groups (Vw) and between groups (Vb) of the extracting data were calculated. The total variance (Vt) was calculated as the sum of Vw and Vb (Vw + Vb). The reliability of each parameter (PRx or wPRx) was then calculated as Vb / Vt.

#### Outcome analysis

To analyze the relationship between PRx/wPRx and patient outcome, the averaged values of each parameter were calculated across the whole monitoring period of each patient. The Glasgow Outcome Scale (GOS) was used to assess patients’ outcome at 6 months, collected by the clinical research nurses in 3 ways: (1) through the hospital record, if the patient died in ward; (2) by patients’ hospital visits to the rehabilitation clinic center after 6-month admission; or (3) by phone interview, answered either by the patient himself/herself or their relatives. The GOS score was scaled into 5 categories: dead, vegetative state (meaning the patient is unresponsive, but alive), severe disability (conscious, but the patient requires others for daily support due to disability), moderate disability (the patient is independent but disabled), and good recovery (the patient has resumed most normal activities but may have minor residual problems) [27]. For the purpose of the statistical analysis, the patients’ outcomes were dichotomized in 2 ways: favorable outcome (good recovery and moderate disability) versus unfavorable outcome (severe disability, vegetative state, and death) and fatal outcome (death) versus nonfatal outcome (good outcome, moderate disability, severe disability, and vegetative state).

#### CPPopt

In order to test the ability of wPRx to delineate CPPopt, the automatic curve-fitting methodology described in detail by Aries et al [14] was used to estimate CPPopt value based on both wPRx (CPPopt_wPRx) and PRx (CPPopt_PRx) for this cohort of patients with TBI. In summary, the PRx (wPRx) values were divided into CPP bins spanning 5 mmHg [14]; the mean value and SD of PRx (wPRx) in each bin were plotted against the bins’ mean CPP to create an error bar chart showing the relationship between PRx (wPRx) and CPP. Theoretically, this relationship should form a U-shape curve with the CPP value at the lowest (w)PRx representing the best CA, termed CPPopt. The CPPopt was calculated using a 4-hour calculation window with an update every minute by fitting a second-order polynomial to the bin averages of Fisher-transformed PRx values and returning the minimal value from within the part of the curve within the available CPP range (please see Aries et al. for more details) [14]. The first CPPopt curve could be generated when at least 50% of the required data points of (w)PRx were available, i.e., after a minimum of 2 hours of monitoring. We calculated the average value of CPPopt across the whole monitoring period for each patient. In addition, the difference between median CPP and CPPopt were calculated continuously (ΔCPP = median CPP – CPPopt) and averaged for the whole monitoring period. The yield index was used to test the continuity of CPPopt and was calculated as the ratio between the count of valid CPPopt values and the count of CPP values across the whole monitoring period of each patient.

### Statistical analysis

Statistical analyses were performed using the IBM SPSS Statistics (version 21) software. The prespecified analyses were described below:

Cross-relationship between wPRx and PRx was studied using Spearman’s correlation coefficient (r) over the hourly averaged data of all patients (n = 65,443). Independent-samples *t* test was used to analyze the ability of wPRx and PRx in distinguishing different outcome groups (favorable versus unfavorable outcome, fatal versus nonfatal outcome). Results were considered significant with *p* < 0.05. Receiver Operating Characteristic (ROC) curves were used to compare the ability of different CPPopts in distinguishing patient outcome, rendering an area under the ROC curve (AUROC) for each parameter [28]. Bland-Altman plots were used to investigate the agreement between wPRx and PRx for the hourly averaged pooled data of all patients with TBI. Statistical differences between ROC curves were verified using the DeLong's test for 2 correlated ROC curves (R package pROC) [29]. The SDs of CPPopt_PRx and CPPopt_wPRx were calculated across the whole monitoring period (1 value per patient).

Following reviewers’ comments and feedback, more post hoc analyses were added:

The stability of PRx and wPRx (2-hour interval SD), and the reliability of the 2 parameters (calculated as ratio of between-group variance to total variance) were compared using Wilcoxon *t* test. The difference between wPRx and PRx was also analyzed using average value of the whole monitoring period (1 value per patient) through paired sample *t* test. The association of PRx and/or wPRx with the patient outcome was investigated using the multivariable binary logistic regression model with stepwise (forward) selection of variables. Three different sets of variables were used for the logistic regressions: the first group with input of (PRx, Glasgow Coma Scale [GCS], ICP, CPP, age, and gender), the second group with input of (wPRx, GCS, ICP, CPP, age, and gender), and the third group including both PRx and wPRx.

## Results

**Fig 1:**
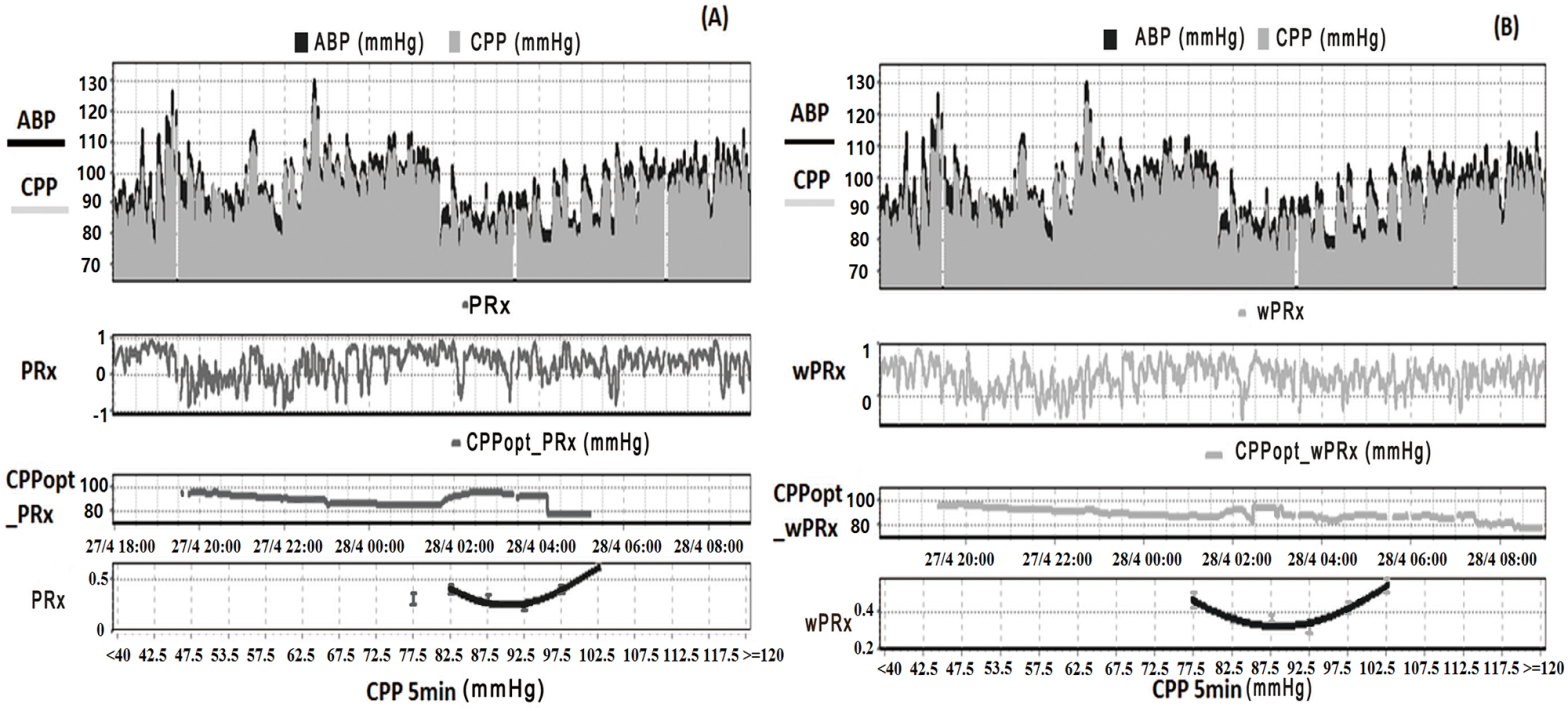
Example of data analysis using ICM+, showing time trends of arterial blood pressure (ABP), cerebral perfusion pressure (CPP), cerebral autoregulation (CA), and optimal CPP (CPPopt). (A) Time trends of ABP, CPP, pressure reactivity index (PRx), and CPPopt according to PRx. (B) Time trends of ABP, CPP, wavelet pressure reactivity index (wPRx), and CPPopt according to wPRx. The U-shape curves at the bottom panels were used to determine automated CPPopt with a curve-fitting method. The (w)PRx-CPP plot was created using CPP 5-minute mean value as x-axis, with averaged (w)PRx values in CPP bins spanning 5 mmHg as y-axis.

The group of patients included 130 females and 385 males, with their characteristics described in Table 1. Their mean age was 38.4 ± 16 (mean ± SD) years old, median GCS score was 7 (interquartile range [IQR]: 3–9). The GCS and GOS score were missing in 34 and 73 patients, respectively. The average ABP and ICP of this cohort was 94.8 ± 15.3 mmHg and 16.2 ± 12.2 mmHg, respectively. Average CPP was 78.6 ± 15.8 mmHg. For the outcome analysis, patients with a missing GOS score were excluded. The outcome was distributed as follows: good recovery, *n* = 75 (17.0%), moderate disability, *n* = 117 (26.5%), severe disability, *n* = 142 (32.1%); persistent vegetative state, n = 11 (2.5%); and death, n = 97 (21.9%). The mean recording time per patient after artifact removal was 118.6 hours (range from 1 hour to 536 hours). Time trends of ABP, CPP, PRx, and wPRx are shown in Fig 1.

**Table 1:** Patient demographics, clinical variables, and outcome.

	*N*	Age	GCS	ABP	ICP	CPP	PRx	wPRx
**Death**	97	44.6 ± 18.2	6.2 (IQR: 3–8)	94.2 ± 14.0	18.7 ± 9.7	75.5 ± 18.8	0.15 ± 0.19	0.37 ± 0.16
**Vegetative state**	11	39.6 ± 17.3	6.6 (IQR: 3–9)	89.0 ± 13.5	15.9 ± 9.0	73.1 ± 10.0	0.05 ± 0.17	0.28 ± 0.12
**Severe disability**	142	39.7 ± 15.6	6.5 (IQR: 4–8)	95.7 ± 21.9	18.2 ± 23.9	77.5 ± 13.4	0.08 ± 0.15	0.28 ± 0.11
**Moderate disability**	117	35.5 ± 16	7.6 (IQR: 4–10)	95.9 ± 29.8	15.8 ± 15.7	80.1 ± 29.4	0.05 ± 0.13	0.24 ± 0.11
**Good recovery**	75	34.1 ± 16	7.5(IQR: 5–10)	92.9 ± 9.7	15.6 ± 10.6	77.3± 12.1	0.01 ± 0.12	0.21 ± 0.10
**Total**	515 (73 GOS missing)	38.4 ± 16	7 (IQR: 3–9)	94.8 ± 15.3	16.2 ± 12.2	78.6 ± 15.8	0.07 ± 0.16	0.28 ± 0.13

ABP, arterial blood pressure; CPP, cerebral perfusion pressure; GCS, Glasgow Coma Scale; GOS, Glasgow Outcome Scale; ICP, intracranial pressure; IQR, interquartile range; M/F, males/females; PRx, pressure reactivity index; SD, standard deviation; wPRx, wavelet pressure reactivity index.

Values are shown as mean ± SD or median and interquartile region. ABP, ICP, CPP, PRx, and wPRx were averaged in each patient over the whole monitoring period.

### Relationship between PRx and wPRx

**Fig 2:**
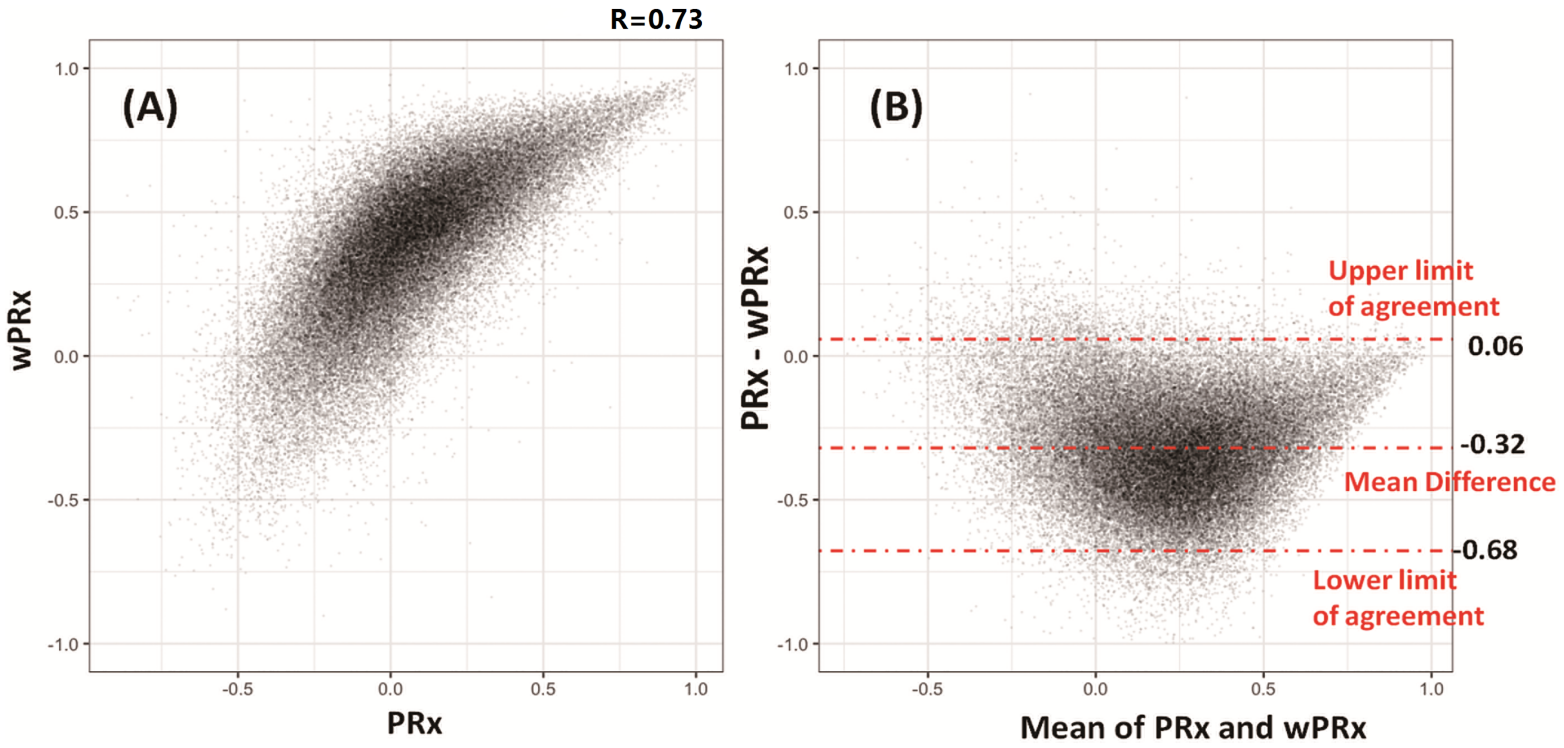
Relationship and agreement between pressure reactivity index (PRx) and wavelet pressure reactivity index (wPRx). (A) There was a strong positive, nonlinear relationship between PRx and wPRx. (B) Bland-Altman plot showing disagreement of the 2 parameters, with a tendency for a positive wPRx-PRx difference.

The data used for relationship analysis between PRx and wPRx were hour-by-hour average data of all the patients, with 65,443 data points in total. Fig 2A shows the relationship between PRx and wPRx. There was a significant positive, nonlinear relationship between the 2 parameters (r = 0.73, Fig 2A). Fig 2B shows a degree of disagreement between the 2 measures, with a tendency for a positive wPRx-PRx difference (the data points do not lie around the y = x line in 2A, and there is a clear "U" shape to 2B, 95% CI on the limits of agreement: −0.68–0.06). The paired *t* test of the average value of wPRx and PRx showed significant difference between the 2 parameters (0.08 ± 0.16 for PRx versus 0.28 ± 0.14 for wPRx, *p* < 0.001). The stability of PRx and wPRx showed a significantly lower 2-hour interval SD with wPRx than with PRx (0.19 ± 0.07 for wPRx and 0.30 ± 0.13 for PRx, *p* < 0.001). The reliability analysis of the 2 methods (PRx and wPRx) showed a significantly increased comparison to PRx (ratio of between-hour variance to total variance, wPRx 0.957 ± 0.0032 versus PRx and 0.949 ± 0.047 for PRx, *p* < 0.001).

### Outcome analysis

**Fig 3:**
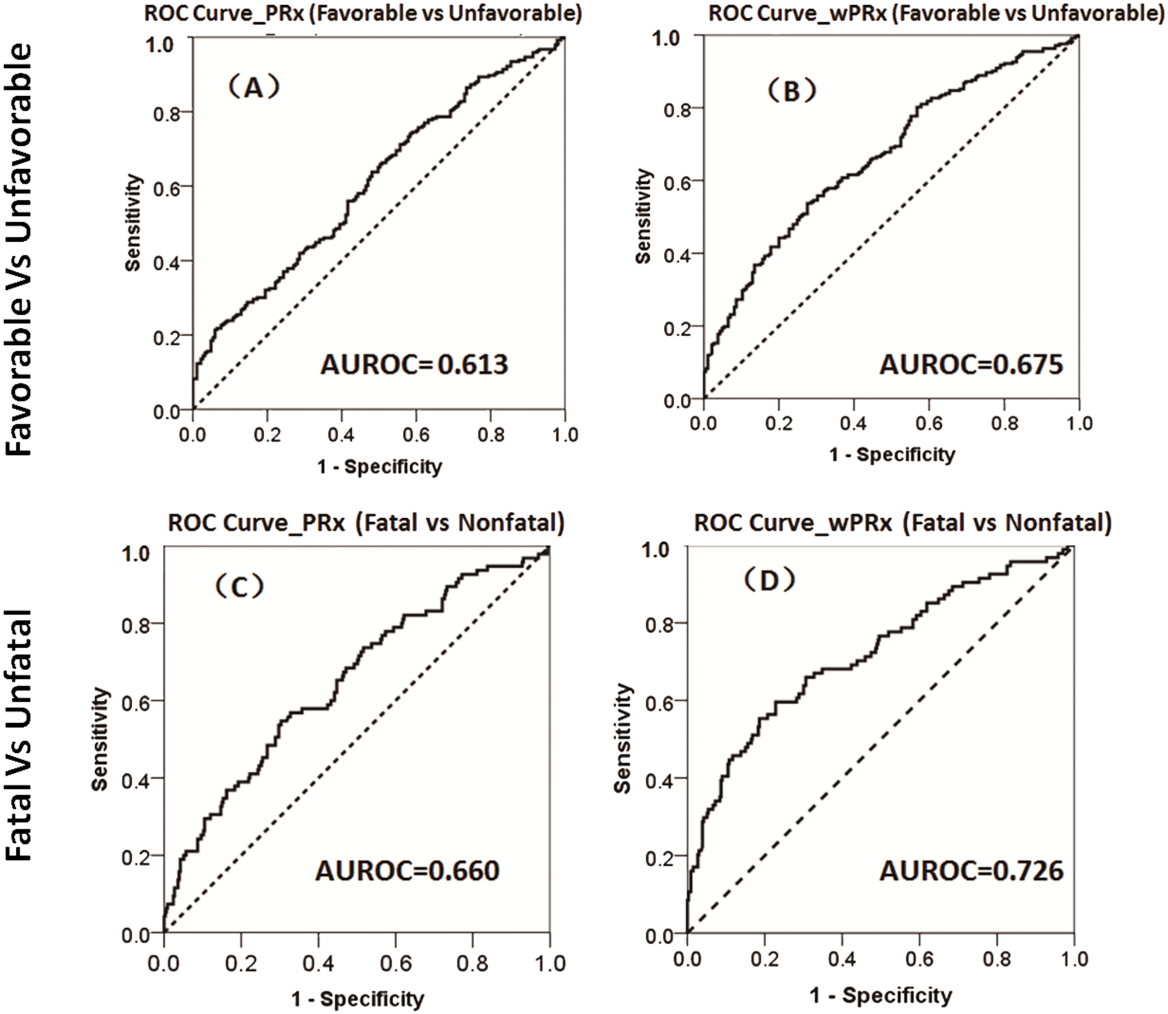
The ability of pressure reactivity index (PRx) and wavelet pressure reactivity index (wPRx) in distinguishing different patient outcome demonstrated by receiver operating characteristic (ROC) curve. (A) ROC curve of PRx in distinguishing the favorable group and unfavorable group. (B) ROC curve of wPRx in distinguishing the favorable and unfavorable groups. (C) ROC curve of PRx in distinguishing the fatal and nonfatal groups. (D) ROC curve of wPRx in distinguishing the fatal and nonfatal groups. AUROC, area under the ROC curve. *N* = 515 (the Glasgow Outcome Scale [GOS] scores were missing in 73 patients), with outcome distributed as follows: good recovery, *n* = 75 (17.0%); moderate disability, *n* = 117 (26.5%); severe disability, *n* = 142 (32.1%); persistent vegetative state, *n* = 11 (2.5%); and death, *n* = 97 (21.9%).

There was a significant difference in both PRx and wPRx for patients with favorable outcome compared to those with unfavorable outcome (Table 2). Mean (±SD) PRx was 0.03 ± 0.13 in the favorable outcome group and 0.10 ± 0.17 in the unfavorable group (*p* < 0.001, AUC = 0.613, Table 2, Fig 3A); wPRx was significantly different between favorable outcome and unfavorable outcome groups (0.31 ± 0.17 versus 0.42 ± 0.18, *p* < 0.001, AUC = 0.675, Fig 3B). The DeLong’s test showed a significant difference between the 2 ROC curves of PRx and wPRx (z = 3.6, *p* = 0.0002).

**Table 2:** The ability of PRx/wPRx in distinguishing favorable and unfavorable patient outcomes.

Index	Mean value of unfavorable outcome	Mean value of favorable outcome	*p*	AUROC	95% Confidence interval	
					Lower bound	Upper bound
PRx (mean ± SD)	0.10 ± 0.17	0.03 ± 0.13	<0.001	0.613	0.561	0.666
wPRx (mean ± SD)	0.42 ± 0.18	0.31 ± 0.17	<0.001	0.675	0.626	0.725

*p* < 0.05 was considered to be significant. The number was expressed as mean ± SD.

AUROC, area under receiver operating characteristic (ROC) curve; PRx, pressure reactivity index; SD, standard deviation; wPRx, wavelet pressure reactivity index.

For the fatal and nonfatal group, PRx and wPRx also showed significant differences between the 2 groups (Table 3). Mean (±SD) PRx was 0.05 ± 0.14 in the nonfatal outcome group and 0.15 ± 0.19 in the fatal group (*p* < 0.001, AUC = 0.66, Fig 3C); wPRx was significantly increased from survival to mortality (0.34 ± 0.17 versus 0.48 ± 0.19, *p* < 0.001, AUC = 0.73, Fig 3D). The DeLong’s test of the 2 ROC curves showed better performance with wPRx than PRx in distinguishing the 2 groups (z = 3.0, *p* = 0.003).

**Table 3:** The ability of PRx/wPRx in distinguishing fatal and nonfatal outcomes.

Index	Mean value of fatal outcome	Mean value of nonfatal outcome	*p*	AUROC	95% Confidence interval	
					Lower bound	Upper bound
PRx (mean ± SD)	0.15 ± 0.19	0.05 ± 0.14	<0.001	0.660	0.597	0.722
wPRx (mean ± SD)	0.48 ± 0.19	0.34 ± 0.17	<0.001	0.726	0.665	0.786

*p* < 0.05 was considered a significant difference between 2 outcome groups. The number was expressed as mean ± SD.

AUROC, area under receiver operating characteristic (ROC) curve; PRx, pressure reactivity index; SD, standard deviation; wPRx, wavelet pressure reactivity index.

The multivariable binary logistic regression model showed that GCS, age, ICP, and PRx/wPRx had a statistically significant effect on mortality as well as on unfavorable outcome prediction (Table 4). If both PRx and wPRx are put into the same model, however, only wPRx retained a significant association with mortality outcomes at the expense of PRx (Table 4). A similar result was also found with unfavorable outcome (Table 5).

**Table 4:** Multiple logistic regression models to predict mortality at 6 months.

Variable	Adjusted OR	95%	*p*	−2 log likelihood
**Model using PRx and Gender, Age, GCS, ICP**				
**PRx**	20.84	2.75–157.96	0.003	332
Gender	0.61	0.31–1.20	0.15	
Age	1.035	0.95–0.98	0.000	
GCS	0.89	0.82–0.97	0.009	
ICP	1.034	1.00–1.07	0.028	
**Model using wPRx and Gender, Age, GCS, ICP**				
**wPRx**	87.03	11.59–635.6	0.000	329
Gender	0.75	0.37–1.49	0.41	
Age	1.04	1.02–1.06	0.000	
GCS	0.88	0.80–0.96	0.005	
ICP	1.03	0.99–1.06	0.12	
**Model using PRx, wPRx and Gender, Age, GCS, ICP**				
**wPRx**	182.71	9.74–3,426.4	0.000	329
**PRx**	0.344	0.017–6.97	0.487	
Gender	0.763	0.38–1.53	0.445	
Age	1.04	1.02–1.06	0.000	
GCS	0.88	0.80–0.96	0.004	
ICP	1.03	0.992–1.06	0.144	

*p* < 0.05 was considered to be significant.

GCS, Glasgow Coma Scale; ICP, intracranial pressure; OR, odds ratio; PRx, pressure reactivity index; wPRx, wavelet pressure reactivity index.

**Table 5:** Multiple logistic regression models to predict unfavorable outcome at 6 months.

Variable	Adjusted OR	95%	*p*	−2 log likelihood
**Model using PRx and Gender, Age, GCS, ICP**				
**PRx**	13.73	2.087–90.37	0.006	421.6
Gender	0.98	0.57–1.70	0.949	
Age	1.034	1.02–1.05	0.000	
GCS	0.864	0.80–0.93	0.000	
ICP	1.03	1.001–1.068	0.044	
**Model using wPRx and Gender, Age, GCS, ICP**				
**wPRx**	27.77	5.60–137.77	0.000	420.4
Gender	1.12	0.64–1.96	0.692	
Age	1.035	1.02–1.05	0.000	
GCS	0.86	0.80–0.93	0.000	
ICP	1.02	0.99–1.05	0.208	
**Model using PRx, wPRx and Gender, Age, GCS, ICP**				
**wPRx**	39.41	3.97–390.88	0.002	420.4
**PRx**	0.56	0.036–8.553	0.674	
Gender	1.13	0.65–1.99	0.663	
Age	1.04	1.02–1.05	0.000	
GCS	0.86	0.80–0.93	0.000	
ICP	1.02	0.99–1.05	0.229	

*p* < 0.05 was considered to be significant.

GCS, Glasgow Coma Scale; ICP, intracranial pressure; OR, odds ratio; PRx, pressure reactivity index; wPRx, wavelet pressure reactivity index.

### CPPopt analysis

**Fig 4:**
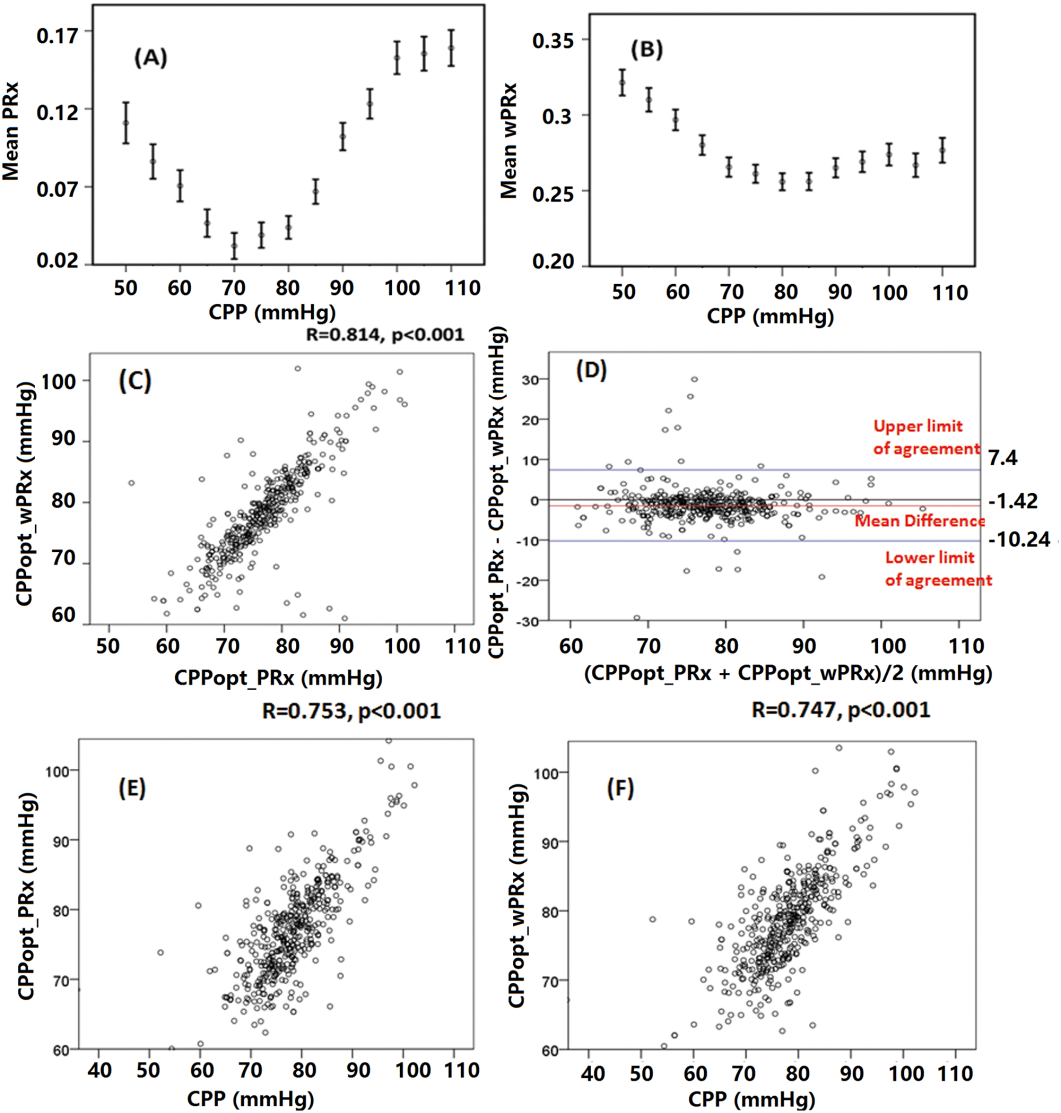
Average cerebral autoregulation (CA) parameters in cerebral perfusion pressure (CPP) bins and the relationship between different optimal CPPs (CPPopts) and between CPP and CPPopt. (A) Pressure reactivity index (PRx) versus CPP plot for the whole cohort. (B) Wavelet pressure reactivity index (wPRx) versus CPP plot for all patients. (C) The relationship between CPPopt calculated according to wPRx (CPPopt_wPRx) and CPPopt based on PRx (CPPPopt_PRx). (D) Bland-Altman plot of CPPopt_wPRx and CPPopt_PRx, showing high agreement of the 2 parameters. (E) CPPopt_PRx versus mean CPP. (F) CPPopt_wPRx versus mean CPP. Each data point in Fig 4C–4F represents the average value of each patient across the whole monitoring period (*n* = 515).

Fig 4 shows the relationship between averaged PRx or wPRx versus CPP values for 515 patients with TBI across the whole monitoring period (Fig 4A and 4B). Both parameters showed a U-shaped relationship with the lowest points at CPP value of between 70 mmHg and 80 mmHg. There was a strong, linear relationship between the 2 CPPopts (r = 0.81, *p* < 0.001, CI: 10.24–7.4, Fig 4C). The Bland-Altman showed high agreement between the 2 CPPopts methods (Fig 4D), with a small overall bias. A significant positive relationship existed between CPPopt_wPRx and mean CPP (r = 0.747, *p* < 0.001, Fig 4F), as well as between CPPopt_PRx and CPP (*r* = 0.753, *p* < 0.001, Fig 4E).

Overall determination of an individual CPPopt across the whole time was possible in 502 patients according to wPRx or PRx. The CPPopt yield was significantly increased by using wPRx (CPPopt_PRx 53.2% ± 20% versus CPPopt_wPRx 59.6% ± 27%, *p* < 0.001). The standard deviation of CPPopt_PRx and CPPopt_wPRx across the whole monitoring period was 8.45 ± 2.90 and 7.05 ± 3.78, respectively (*p* < 0.001).

**Fig 5:**
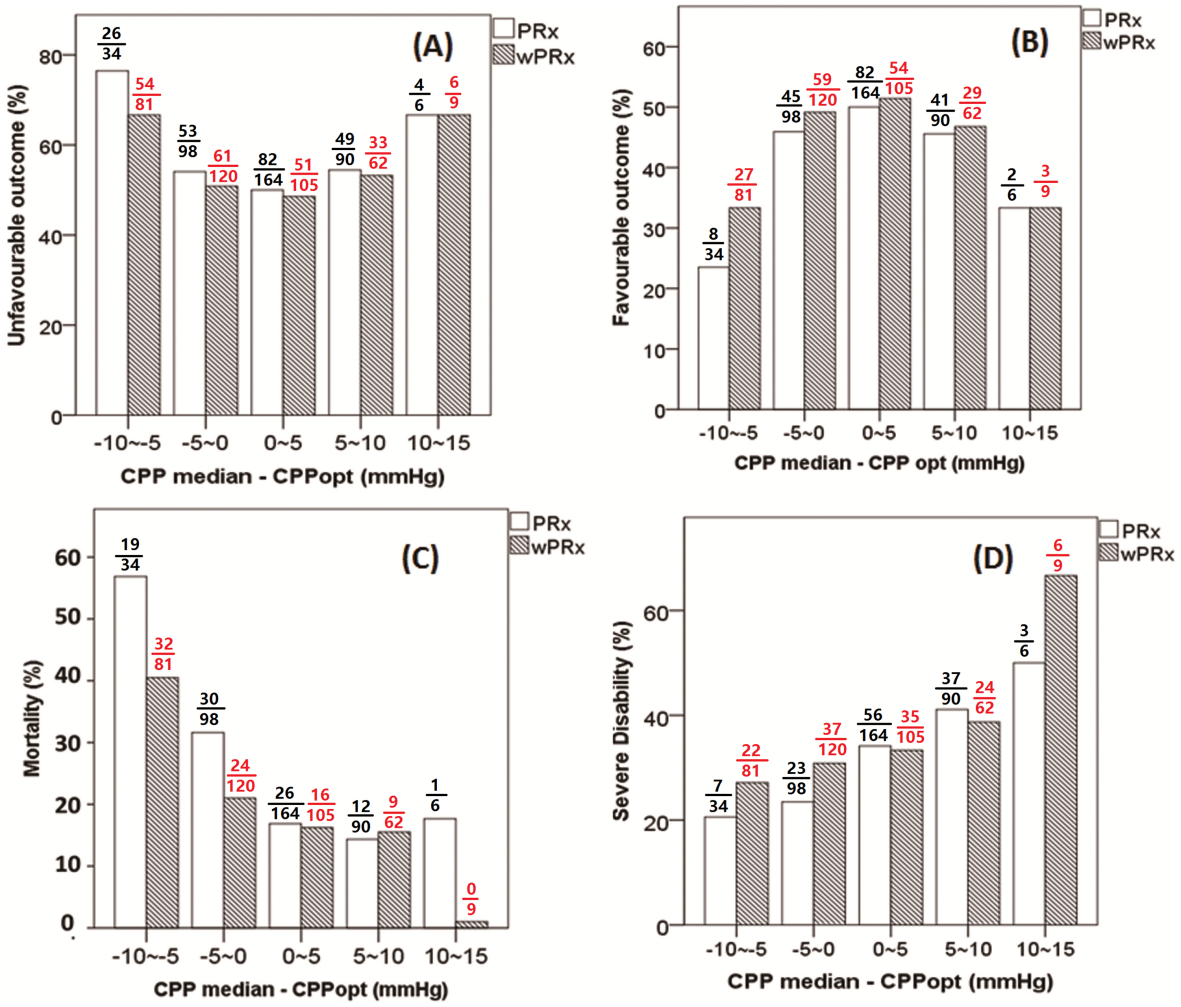
Graphs of the relationship between patient outcome and ΔCPP according to pressure reactivity index (PRx) (white bar) and wavelet pressure reactivity index (wPRx) (striped bar). (A) A U-shape curve demonstrating that the smallest incidence of unfavorable outcome was associated with median cerebral perfusion pressure (CPP) around optimal CPP (CPPopt). (B) There was an asymmetrical inversed U-shape curve between favorable outcome and ΔCPP. (C) The mortality increases steadily when median CPP is increasingly below CPPopt. (D) Severe disability increased while CPP median is above CPPopt. The denominator represents the total patient number in each bar, and the numerator indicates the patient in each outcome group. ΔCPP, the difference between median CPP and CPPopt.

**Fig 6:**
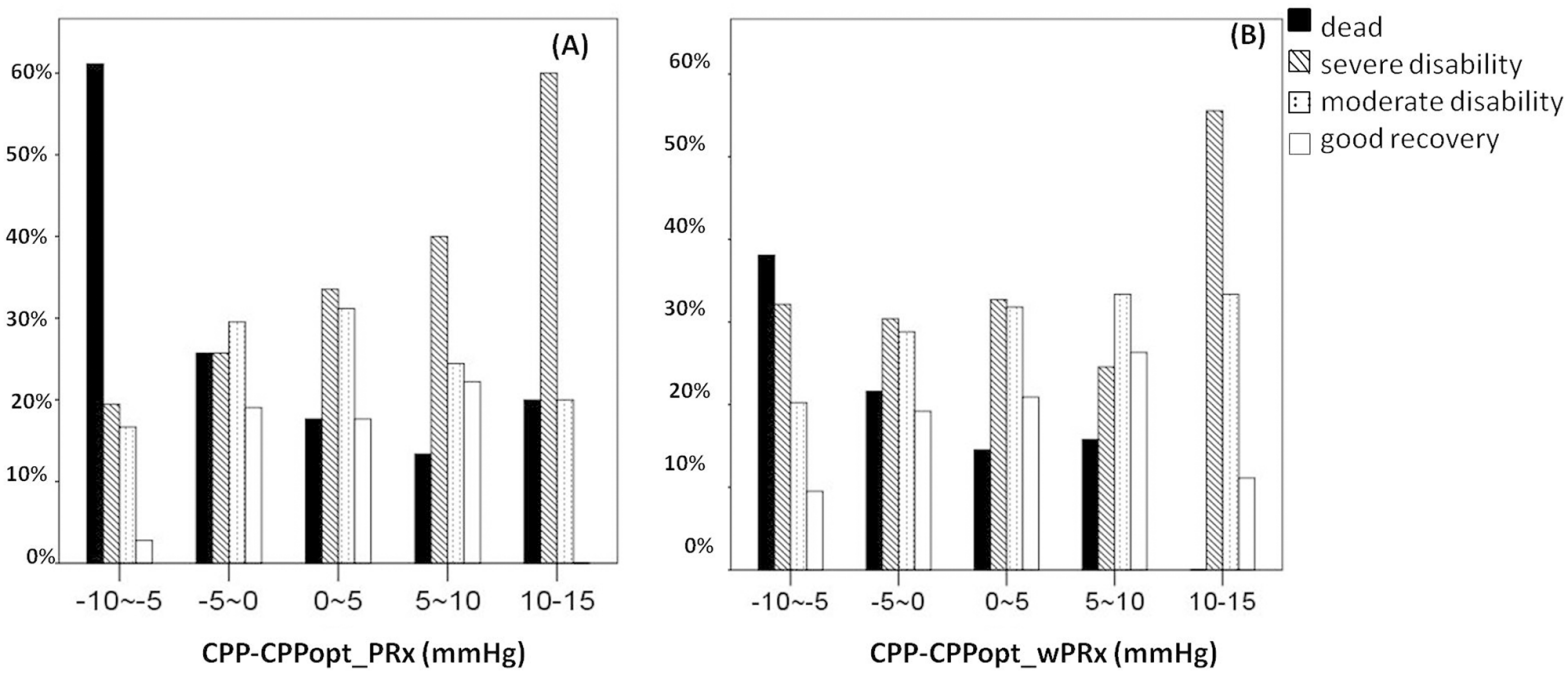
Distribution of Glasgow Outcome Scale (GOS) Score (%) versus the binned difference between the median cerebral perfusion pressure (CPP) and optimal CPP (CPPopt). (A) Distribution of GOS Score versus the binned difference between median CPP and CPPopt _PRx; (B) Distribution of GOS Score versus the binned difference between median CPP and CPPopt _wPRx. CPPopt_PRx, optimal cerebral perfusion pressure according to pressure reactivity index (PRx); CPPopt_wPRx, optimal cerebral perfusion pressure according to wavelet PRx.

Fig 5 demonstrates the relationship between favorable outcome, unfavorable outcome, mortality rate, severe disability rate, and ΔCPP. The mortality increased steadily with the median CPP shifting below the threshold of CPPopt both according to wPRx and PRx (Fig 5C). An inverse “U” shape curve with the highest favorable outcome rate appeared at the smallest difference between CPP and CPPopt is seen in Fig 5B. To the contrary, the unfavorable outcome showed a rate increasing below or above CPPopt (Fig 5A). Severe disability rate was increased, while median CPP is above CPPopt (Fig 5D). Fig 6 shows the distribution of GOS Score (%) versus binned difference between the median CPP and CPPopt_PRx or median CPP and CPPopt_wPRx bins for the whole monitoring period.

### Age and ICP

**Fig 7:**
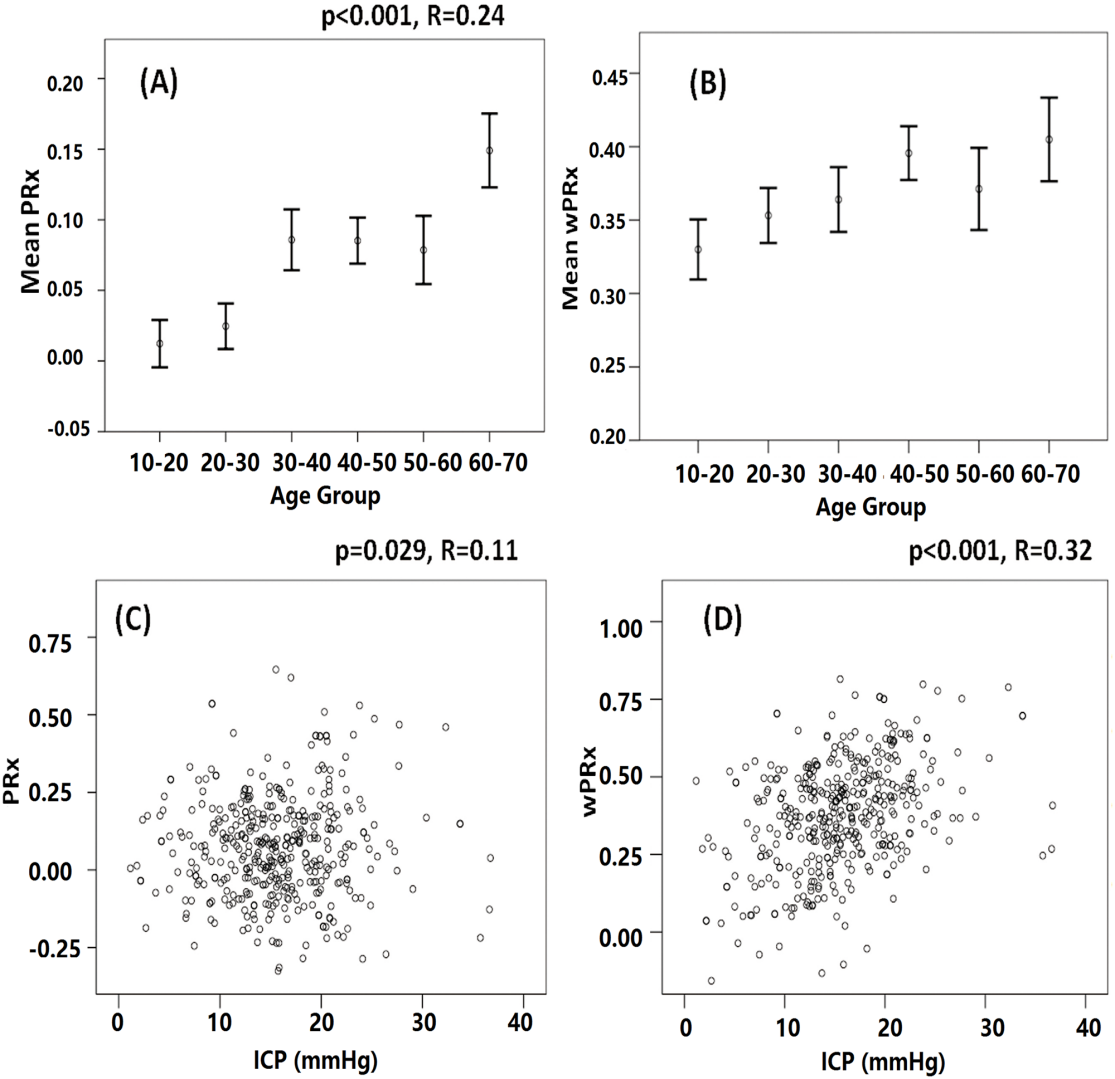
The relationship between cerebral pressure reactivity parameters, age, and intracranial pressure (ICP). (A) Cerebral pressure reactivity seems to have deteriorated with the increase of age, shown by increased pressure reactivity index (PRx). (B) Wavelet pressure reactivity index (wPRx) also showed a slight increase while the age was increased. (C) PRx was linearly related with ICP. (D) A linear relationship existed between wPRx and ICP.

There was a significant relationship between PRx and age (r = 0.24, *p* < 0.001) and also between wPRx and age (Fig 7, r = 0.124, *p* = 0.01) in this cohort of 515 patients with TBI. Cerebral pressure reactivity seems to have deteriorated with the increase of age, shown by increased PRx and wPRx. wPRx showed a stronger relationship with ICP (r = 0.32, *p* < 0.001) than PRx (r = 0.11, *p* = 0.029).

## Discussion

The wavelet transform is a time—frequency method that facilitates more accurate analysis of biological signals containing components that are nonstationary [30]. In this study, we have introduced and developed a wavelet-based methodology of continuous assessment of CA for 515 patients with TBI, termed wPRx, and compared it with a widely used parameter, PRx. The ability of the new parameter to distinguish different patient outcomes was also examined. There were 5 principal findings in this study: (1) a close relationship existed between PRx and wPRx, (2) wPRx showed more stable trends, (3) wPRx demonstrated better performance than PRx in terms of distinguishing different patient outcomes, (4) wPRx makes CPPopt estimation more continuous, and (5) pressure reactivity deteriorated while the age is increased.

First, the study showed a close relationship between wPRx and the established PRx index. For PRx, a negative correlation indicates a pressure-active vascular bed with preserved cerebrovascular reactivity, whereas a positive correlation indicates a pressure-passive vascular bed with impaired reactivity [30]. While for wPRx, bigger wPRx values reflect smaller phase shifts between fluctuations in ABP and ICP, indicating direct changes in ICP following ABP because of impaired cerebrovascular function. The fact that we have achieved such a correlation between the 2 indices might suggest that the PRx, the correlation coefficient index, actually also reflects the phase shift between 2 waves.

Second, we found that despite similar performance from both indices, the wPRx approach seemed to produce more stable and reliable time trends, which was reflected by its smaller standard deviation and higher reliability index compared with PRx. The improved precision in wPRx versus PRx may be explained by the robustness of the wavelet method for determining phase shift from nonstationary signals [26,31]. PRx is just a simple correlation coefficient parameter, which takes all the information into calculation without removing any uncertain noise. However, wPRx, by using wavelet coherence as a filter, guarantees a reliable relationship between ABP and ICP. By removing points that represent noise, wPRx produces a more stable estimation of pressure reactivity than traditional PRx.

Moreover, the relationship between PRx and wPRx showed that at PRx close to 0, wPRx varies widely (Fig 2). As mentioned above, calculated as a correlation coefficient, PRx around 0 can either mean moderate autoregulation but not fully engaged, or this could be due to strong extraneous noise, which totally destroys the correlation between the 2 variables. On the other hand, wPRx with high coherence as a guarantee of reliable relationship between ABP and ICP will not be influenced by those effects and thus reflects true autoregulation.

The third main finding of this retrospective study was that wavelet demonstrated better performance than PRx in distinguishing different patient outcomes: favorable versus unfavorable outcome and fatal versus nonfatal outcome. The AUROC of wPRx was significantly larger than that of PRx in distinguishing 2 outcome groups. Multiple logistic regression models including one CA parameter (either wPRx or PRx) demonstrated that each parameter was predictive of outcome; however, when both wPRx and PRx were included in the same model, only wPRx was significant. This indicates that although the 2 are measuring the same phenomenon, wPRx is superior to PRx in terms of prognosis.

Fourth, in CPPopt calculation, wPRx makes CPPopt estimation more continuous, which is shown through higher yield than CPPopt according to PRx. Patient outcome correlated similarly with the difference between median CPP and CPPopt_ wPRx as the difference between median CPP and CPPopt_PRx. Mortality was associated with relative hypoperfusion (CPP < CPPopt_wPRx), severe disability with hyperperfusion (CPP > CPPopt_wPRx); favorable outcome was associated with a smaller difference between CPP and CPPopt_wPRx. For the whole cohort of patients with TBI, the asymmetrical “U”-shape curve created by plotting wPRx versus CPP bins (Fig 4B) might suggest that CPP below the averaged optimal level can impair cerebral pressure reactivity more severely than a CPP above the averaged optimal level. This might explain why mortality was more commonly increased while CPP was below CPPopt (Fig 5C).

The fifth finding of this research was an age-related deterioration in CA, which indicates that perfusion pressure autoregulation in older patients with TBI no longer performs as efficiently as in younger patients. This relationship was captured by both PRx and wPRx. Normal aging is associated with many well-recognized changes in the cardiovascular system, such as increases in systolic blood pressure, decreases in systemic artery compliance, cardiac baroreceptor sensitivity, and decrease of cortical cerebral blood flow [35–38]. Interestingly, in healthy volunteers, several studies have shown no relationship between cerebrovascular CA and age using ABP as input and flow velocity as output [32,33].

### Limitations

Three limitations should be noted:

Although this study has a large sample size, it is a retrospective analysis. The data were preprocessed before calculating wPRx, PRx, and CPPopt, with artifacts removed manually. Although, this retrospective analysis confirmed that patients whose median CPP is closer to their determined CPPopt seem to have better clinical outcomes, a prospective validation of these findings is essential, to determine whether CPPopt can be used in prognostic determination or CPP-target guiding treatment to improve patient outcome. Moreover, according to the retrospective study, wPRx showed more stable performance. A randomized controlled trial of CPPopt targeting is essential to suggest which (wPRx or PRx) will be the best technique for pressure reactivity assessment.

Although the CPPopt yield was significantly increased by using wPRx, we were only able to estimate CPPopt in around 60% of the monitoring time. Further improvements to the algorithm need to be introduced, as, for example, suggested by Depreitere et al. [34], in order to increase the yield and make the method clinically more practical.

This study already demonstrated a close relationship between PRx and wPRx; it would be very interesting to compare the wPRx calculations to an autoregulation index derived from an alternative measure of CA (for example, intracranial blood vessel flow velocity—derived measures in humans) or more direct measures of CA in an animal model.

## Conclusion

This study introduces a new, wavelet-based method for cerebral pressure reactivity assessment in patients with TBI. Based on these findings, wPRx is stable in time, yields consistent CPPopt recommendation, and, importantly, has a strong relationship with patient outcome.

## Supporting information

S1 Checklist STROBE checklist. (DOCX)

S1 Appendix Wavelet Transform algorithm. (DOCX)

S1 Research Plan Research plan. (PDF)

## Abbreviations

ABP: arterial blood pressure
AUROC: area under the receiver operating characteristic curve
CA: cerebral autoregulation
CPP: cerebral perfusion pressure
CPPopt: optimal CPP
GCS: Glasgow Coma Scale
GOS: Glasgow Outcome Scale
ICP: intracranial pressure
IQR: interquartile range
PRx: pressure reactivity index
SD: standard deviation
TBI: traumatic brain injury
wPRx: wavelet pressure reactivity index
WTP: wavelet transform phase shift
WTC: wavelet transform coherence
