# Supplementary material for: Cerebrovascular pressure reactivity monitoring using wavelet analysis in traumatic brain injury patients: A retrospective study
Source: PLoS Med. 2017 Jul 25;14(7):e1002348. doi: 10.1371/journal.pmed.1002348 (PMC5526510; doi:10.1371/journal.pmed.1002348)
Supplement: S1 Checklist — (DOCX) [file pmed.1002348.s001.docx]

**S1 STROBE Checklist**

|  | Item No | Recommendation | Section paragraph(s) |
| --- | --- | --- | --- |
| **Title and abstract** | 1 | **(*a*) Indicate the study’s design with a commonly used term in the title or the abstract.**  The study design is included in the title of the paper: ‘Cerebrovascular pressure reactivity monitoring using wavelet analysis in traumatic brain injury patients: A retrospective study’ . | - |
|  |  | **(b) Provide in the abstract an informative and balanced summary of what was done and what was found.**  We have provided this information in the Methods and Findings sections of our abstract:  Five hundred and fifteen patients with TBI admitted in Addenbrooke’s Hospital, United Kingdom (March 23rd, 2003 through December 9th, 2014), with continuous monitoring of arterial blood pressure (ABP) and intracranial pressure (ICP), were retrospectively analyzed to calculate the traditional PRx and a novel wavelet transform based wPRx. wPRx was calculated by taking the cosine of the wavelet transform phase-shift between ABP and ICP. A time trend of CPPopt was calculated using an automated curve-fitting method that determined the cerebral perfusion pressure (CPP) at which the pressure reactivity (PRx or wPRx) was most efficient (CPPopt_PRx and CPPopt_wPRx, respectively).  There was a significantly positive relationship between PRx and wPRx (r = 0.73), and wavelet wPRx was more reliable in time (ratio of between-hour variance to total variance, wPRx 0.957 ± 0.0032 versus PRx and 0.949 ± 0.047 for PRx, p = 0.002). The 2-hour interval standard deviation of wPRx (0.19 ± 0.07) was smaller than that of PRx (0.30 ± 0.13, p < 0.001). wPRx performed better in distinguishing between mortality and survival (the area under the receiver operating characteristic [ROC] curve [AUROC] for wPRx was 0.73 versus 0.66 for PRx, p = 0.003). The mean difference between the patients’ CPP and their CPPopt was related to outcome for both calculation methods. There was a good relationship between the 2 CPPopts (r = 0.814, p < 0.001). CPPopt_wPRx was more stable than CPPopt_PRx (within patient standard deviation 7.05 ± 3.78 versus 8.45 ± 2.90; p < 0.001). | Paragraph 2 in the abstrct |
| Introduction | | |  |
| Background/rationale | 2 | Explain the scientific background and rationale for the investigation being reported | Paragraph 1-4 in the introduction section |
| Objectives | 3 | State specific objectives, including any prespecified hypotheses | Paragraph 5, in the introoduction section |
| Methods | | |  |
| Study design | 4 | Present key elements of study design early in the paper | Paragraph 4-5 in the introduction secton |
| Setting | 5 | Describe the setting, locations, and relevant dates, including periods of recruitment, exposure, follow-up, and data collection | Paragraph 3 and 7 in the methods section |
| Participants | 6 | (*a*) Give the eligibility criteria, and the sources and methods of selection of participants. Describe methods of follow-up | Data sample section |
|  |  | (*b*) For matched studies, give matching criteria and number of exposed and unexposed | NA |
| Variables | 7 | Clearly define all outcomes, exposures, predictors, potential confounders, and effect modifiers. Give diagnostic criteria, if applicable | Outcome analysis paragraph in the methods section |
| Data sources/ measurement | 8* | For each variable of interest, give sources of data and details of methods of assessment (measurement). Describe comparability of assessment methods if there is more than one group | Paragraph 3 and 8 in the Methods section |
| Bias | 9 | Describe any efforts to address potential sources of bias | Paragraph 1 in the Results section |
| Study size | 10 | Explain how the study size was arrived at | Paragraph 3 and 8 in the Methods section, and paragraph 1 in results section |
| Quantitative variables | 11 | Explain how quantitative variables were handled in the analyses. If applicable, describe which groupings were chosen and why | Paragraph 3 in methods section |
| Statistical methods | 12 | (*a*) Describe all statistical methods, including those used to control for confounding | Statistical Analysis part in Methods section |
|  |  | (*b*) Describe any methods used to examine subgroups and interactions | Paragraph 1-3 in Statistical analysis section |
|  |  | (*c*) Explain how missing data were addressed | Paragraph 1 in Results section |
|  |  | (*d*) If applicable, explain how loss to follow-up was addressed | NA |
|  |  | (*e*) Describe any sensitivity analyses | Paragraph 2 in Statistical Analysis section |
| Results | | |  |
| Participants | 13* | (a) Report numbers of individuals at each stage of study—eg numbers potentially eligible, examined for eligibility, confirmed eligible, included in the study, completing follow-up, and analysed | Paragraph 1, table 1 in Results section |
|  |  | (b) Give reasons for non-participation at each stage | Paragraph 1 in Results section |
|  |  | (c) Consider use of a flow diagram | NA |
| Descriptive data | 14* | (a) Give characteristics of study participants (eg demographic, clinical, social) and information on exposures and potential confounders | Table 1 |
|  |  | (b) Indicate number of participants with missing data for each variable of interest | Paragraph 1 and table 1 in results section |
|  |  | (c) Summarise follow-up time (eg, average and total amount) | Paragraph 1 in Results section |
| Outcome data | 15* | Report numbers of outcome events or summary measures over time | Table 1 |
| Main results | 16 | (*a*) Give unadjusted estimates and, if applicable, confounder-adjusted estimates and their precision (eg, 95% confidence interval). Make clear which confounders were adjusted for and why they were included | Paragraph 2-4, 6,7 in the results section |
|  |  | (*b*) Report category boundaries when continuous variables were categorized | NA |
|  |  | (*c*) If relevant, consider translating estimates of relative risk into absolute risk for a meaningful time period | NA |
| Other analyses | 17 | Report other analyses done—eg analyses of subgroups and interactions, and sensitivity analyses | Paragraph 5,8,9 in results section |
| Discussion | | |  |
| Key results | 18 | Summarise key results with reference to study objectives | Paragraph 1-7 in discussion section |
| Limitations | 19 | Discuss limitations of the study, taking into account sources of potential bias or imprecision. Discuss both direction and magnitude of any potential bias | Limitations section |
| Interpretation | 20 | Give a cautious overall interpretation of results considering objectives, limitations, multiplicity of analyses, results from similar studies, and other relevant evidence | Conclusion |
| Generalisability | 21 | Discuss the generalisability (external validity) of the study results | Paragraph 1 in limitations section |
| Other information | | |  |
| Funding | 22 | Give the source of funding and the role of the funders for the present study and, if applicable, for the original study on which the present article is based.  We have included this information in our financial disclosure:   - ICM+ Software is licensed by Cambridge Enterprise, Cambridge, UK, http://www.neurosurg.cam.ac.uk/icmplus/. Peter Smielewski and Marek Czosnyka have a ﬁnancial interest in a fraction of the licensing fee. - Xiuyun Liu is recipient of Bill Gates Scholarship (University of Cambridge) - Joseph Donnelly is funded by the Woolf Fisher Trust, NZ (Woolf Fisher Scholarship). - Danilo Cardim is supported by a Cambridge Commonwealth, European & International Trust Scholarship, University of Cambridge. | - |

*Give information separately for exposed and unexposed groups.

**Note:** Information on the STROBE Initiative is available at http://www.strobe-statement.org.
