# Supplementary material for: Cerebrovascular pressure reactivity monitoring using wavelet analysis in traumatic brain injury patients: A retrospective study
Source: PLoS Med. 2017 Jul 25;14(7):e1002348. doi: 10.1371/journal.pmed.1002348 (PMC5526510; doi:10.1371/journal.pmed.1002348)
Supplement: S1 Appendix — (DOCX) [file pmed.1002348.s002.docx]

## S1 Appendix. Wavelet Transform Algorithm

The wavelet transform is defined as the convolution of a scaled parent wavelet function $\varphi$ with the analysed function g(t),

$W(s,\tau)=\int g\left( t \right)\varphi_{s}\left( t-\tau\right)dt$ (F1)

Morlet wavelet was chosen as the mother wave, defined as

$\varphi_{0}\left( t \right)= \pi^{-\frac{1}{4}}(e^{i2\pi f_{0}t}-e^{-{(2\pi f_{0)}}^{2}/2})e^{-t^{2}/2}$ (F2)

Where f_0_ is the central frequency, t is time. The second term in the brackets is known as the correction term, as it corrects for the non-zero mean of the complex sinusoid of the first term. In practice it becomes negligible and can be ignored while f_0_$\gg0$, in which case, the Morlet wavelet can be written in a simpler form as [1]

$\varphi_{0}\left( t \right)= \pi^{-\frac{1}{4}}e^{i2\pi f_{0}t}e^{-t^{2}/2}$ (F3)

Here the scale s was defined as :

f=f_0_/s=w_0_/2$\pi$s (F4)

where w_0_ is the reference coefficient, which equals central frequency divided by 2$\pi$. W_0_ shifts the balance between frequency resolution and time resolution. Therefore the wavelet function based on the Morlet wavelet function can be expressed as

$\varphi\left( t/s \right)= \pi^{-\frac{1}{4}}e^{iw_{0}t/s}e^{-{(t/s)}^{2}/2}$ (F5)

The CWT of atime series (x_n_, n=1, 2,…, N) with uniform time steps $\delta t$, is defined as the convolution of x_n_ with the scaled and normalized wavelet, which can be described as F6-F7:

$W_{n}(s)=\sum_{n^{'}=0}^{N-1} x_{n^{'}}\varphi^{*}[\frac{(n^{'}-n)\delta t}{s}]$ (F6)

$\varphi[\frac{\left( n^{'}-n \right)\sigma t}{s}]=\left( \frac{\delta t}{s} \right)^{\frac{1}{2}}\varphi_{0}[\frac{(n^{'}-n)\delta t}{s}]$ (F7)

Where $\varphi^{*}$ is the complex conjugate of the normalized wavelet function; n is the time-series index, and $\delta t$ is the sampling time. We define$A=W_{n}\left( S \right){W_{n}(S)}^{*}$ as the wavelet power density, the complex argument of $W_{n}(s)$ can be interpreted as the local phase.

The finite length of the signal resulted in the edge artifacts of WT. It is therefore useful to introduce an index, Cone of Influence (COI), in which the edge effect is significant and the wavelet power for discontinuity at the edge drops by a factor of $e^{-2}$. The edge effect is $s\sqrt{2}$ for Morlet wavelet [2,3]. The points within the edge effct area were removed prior to the phase/coherence point extraction. COI is larger for larger w_0_.

**The cross wavelet transform**

The cross wavelet transform (XWT) of two time series x_n_ and y_n_ is deﬁned as

$W^{XY}=W^{X}W^{Y*}$ (F8)

where * denotes complex conjugation. The complex argument of W^XY^ can be interpreted as the local relative phase shift between x_n_ and y_n_ [3–5].

**Wavelet transform coherence**

The squared wavelet coherence is deﬁned as the squared absolute value of the smoothed cross-wavelet spectrum, normalized by the smoothed wavelet power spectrum of the two signals,

$C_{n}^{2}\left( s \right)= \frac{\left| <W_{n}^{xy}(s)\cdot s^{-1}> \right|^{2}}{<W_{n}^{xx}(s)\cdot s^{-1}><W_{n}^{yy}(s)\cdot s^{-1}>}$ (F9)

where $W_{n}^{xx}$and $W_{n}^{yy}$ are the wavelet spectral density function; $W_{n}^{xy}$ is the cross-wavelet spectrum ; and the angular brackets indicate the smoothing operator. This deﬁnition of the wavelet coherence corresponds with the Fourier-based coherence and maintains its value between 0 and 1.

The smoothing operator is achieved by a convolution in time and scale.

$$S\left( W \right)=S_{scale}\left( S_{time}\left( W_{n}\left( S \right) \right) \right), (F10)$$

where Sscale implies smoothing along the wavelet scale axis and Stime means smoothing in time. The time convolution is performed with a Gaussian $e^{-n\frac{2}{{2s}^{2}}}$, which is the absolute value of the wavelet function in each scale. The time convolution will double the edge artefact to 2s$\sqrt{2}$. The scale convolution is performed by a rectangular window with a length of $\sigma_{j0}\cdot s$, where $\sigma_{j0}=0.6$ is the empirical scale decorrelation length for the Morlet wavelet [6],

${\left\langle W \right\rangle=\left[ {(c_{1}w_{n}\left( s \right)*e^{-n^{2}/2s^{2}})}_{n}*c_{2}\prod(\delta_{j0}s) \right]}_{s}$ (F11)

c1 and c2 are the normalization factors and II is the rectangular function.

**References**

1. Addison P. The Illustrated Wavelet Transform Handbook. Biomedical Instrumentation & Technology. 2002. doi:10.1201/9781420033397

2. Keissar K, Davrath LR, Akselrod S. Coherence analysis between respiration and heart rate variability using continuous wavelet transform. Philos Trans A Math Phys Eng Sci. 2009;367: 1393–1406. doi:10.1098/rsta.2008.0273

3. Grinsted a., Moore JC, Jevrejeva S. Application of the cross wavelet transform and wavelet coherence to geophysical time series. Nonlinear Process Geophys. 2004;11: 561–566. doi:10.5194/npg-11-561-2004

4. Peng TPT, Rowley a. B, Ainslie PN, Poulin MJ, Payne SJ. Wavelet Phase Synchronization Analysis of Cerebral Blood Flow Autoregulation. IEEE Trans Biomed Eng. 2010;57: 960–968. doi:10.1109/TBME.2009.2024265

5. Latka M, Turalska M, Glaubic-Latka M, Kolodziej W, Latka D, West BJ. Phase dynamics in cerebral autoregulation. Am J Physiol Heart Circ Physiol. 2005;289: H2272–H2279. doi:10.1152/ajpheart.01307.2004

6. Torrence C, Compo GP. A Practical Guide to Wavelet Analysis. Bull Am Meteorol Soc. 1998;79: 61–78. doi:10.1175/1520-0477(1998)079<0061:APGTWA>2.0.CO;2
