# Supplementary material for: Cerebrovascular pressure reactivity monitoring using wavelet analysis in traumatic brain injury patients: A retrospective study
Source: PLoS Med. 2017 Jul 25;14(7):e1002348. doi: 10.1371/journal.pmed.1002348 (PMC5526510; doi:10.1371/journal.pmed.1002348)
Supplement: S1 Research Plan — (PDF) [file pmed.1002348.s003.pdf]

Research Plan

Cerebrovascular Pressure Reactivity Monitoring Using Wavelet Analysis in Traumatic Brain Injury Patients

Aim

Introduce a robust method to assess cerebral autoregulation (CA) in TBI patients

Materials

515 TBI patients admitted in Addenbrooke's Hospital, UK with continuous monitoring of arterial blood pressure (ABP) and intracranial pressure (ICP)

Hypothesis

1. Higher wavelet phase shift, (lower wPRx) is related with worse CA; while smaller wavelet phase shift, i.e. higher wPRx, is related with better CA;

2. The wavelet method should demonstrate better performance than the widely used method, pressure reactivity index (PRx), in terms of reliability, stability and distinguishing patient outcome.

3. The wavelet method should show better performance in estimating an optimal cerebral perfusion pressure (CPPopt) for TBI patient than PRx.

1st Step

- Wavelet method is implemented into ICM+ software
- Wavelet coherence threshold decided through 10000 Monte Carlo simulations

2nd Step

- wPRx was calculated as cosine of wavelet phase shift between ABP and ICP
- PRx was calculated as correlation coefficient between ABP and ICP
- CPPopt will be calculated using an automatic curve fitting methodology described in detail by Aries et al 2012.

3rd Step

- Analyze the relationship between wPRx and PRx
- Compare the reliability and stability between PRx and PRx
- Analyze the ability of wPRx and PRx of giving CPPopt recommendation

Research Report

1. Pre-analysis research plan

1) <u>Study Topic:</u>

Cerebrovascular Pressure Reactivity Monitoring Using Wavelet Analysis in Traumatic Brain Injury Patients: A Retrospective Study

2) <u>The main Issue or problem</u>

Monitoring cerebral autoregulation (CA) is especially important for traumatic brain injury (TBI) patients. There are still some problems with current methods for CA assessment. Introducing a more robust method is necessary both in research and clinically.

3) <u>The aim and hypotheses of this study</u>

Wavelet method has been widely applied in analyzing noisy, transient and non-stationary signals. One advantage of wavelet analysis is the ability to perform local analysis and reveal signal features with desired temporal–frequency resolution. Using coherence threshold to ensure a reliable relationship between ABP and ICP, we hypothesize that this method would perform better in CA assessment than a simple time correlation approach (PRx).

<u>Hypothesis I</u>: The wavelet method can be used to assess CA for TBI patients. Higher wavelet phase shift, which results in smaller cosine value of the phase shift (terms as wPRx) is related with worse cerebral autoregulation; while smaller wavelet phase shift, i.e. higher wPRx, is related with better cerebral autoregulation;

Hypothesis II: The wavelet method offers better performance than the widely used method, pressure reactivity index (PRx), in terms of lower variability and distinguishing patient outcome.

Hypothesis III: Estimations of optimal cerebral perfusion pressure (CPPopt) in TBI patients using wPRx results in more stable trend with less discontinuities than using PRx.

4) Methods and materials

515 TBI patients admitted in Addenbrooke's Hospital, UK (March 2003- December 2014), with continuous monitoring of arterial blood pressure (ABP) and intracranial pressure (ICP) were retrospectively analyzed.

The PRx was calculated as a moving Pearson correlation coefficient between 10s averages of ABP and mean ICP, using a 300s data window.

Wavelet wPRx was calculated as cosine of wavelet transform phase shift between ABP and ICP, in the frequency of 0.0067 Hz to 0.05 Hz. Morlet mother wave with the central frequency at 1 Hz was applied and a 500-second window was used. Wavelet transform coherence (WTC) was applied as an indicator of a reliable phase relationship between input and output, decided through Monte Carlo simulations approach.

In order to test the ability of wPRx to delineate the optimal cerebral perfusion pressure (CPPopt), the automatic curve fitting methodology described in detail by Aries et al was used to estimate CPPopt value based on both wPRx (CPPopt_wPRx) and PRx (CPPopt_PRx) for this cohort of TBI patients.

The relationship between PRx and wPRx, the reliability and stability of the two parameters, the ability of PRx and wPRx of giving CPPopt recommendation will be studied. The relationship between PRx or wPRx with patient outcome will also be compared.

2. Research Main Result

The result of this study positively verified the hypotheses listed in the research plan:

Result I: There was a significantly positive relationship between PRx and wPRx (r = 0.73)

Result II: wPRx was more stable in time (2-hour interval standard deviation of wPRx (0.19± 0.07) was smaller than that of PRx (0.30 ± 0.13, p<0.001)). wPRx was more reliable(ratio of between-hour variance to total variance, wPRx 0.957± 0.0032 vs PRx and 0.949 ± 0.047 for PRx, p=0.002). Moreover, wPRx performed better in distinguishing between mortality and survival (AUROC for wPRx was 0.73 vs 0.66 for PRx, p = 0.003).

Result III: CPPopt yield was significantly increased by using wPRx (CPPopt_PRx 53.2% ± 20% vs CPPopt_wPRx 59.6% ± 27%, p<0.001) and CPPopt_wPRx was more stable (within patient standard deviation 7.05 ± 3.78 vs 8.45 ± 2.90; p<0.001).
